# The auditory comprehension changes over time after sport‐related concussion can indicate multisensory processing dysfunctions

**DOI:** 10.1002/brb3.874

**Published:** 2017-11-29

**Authors:** Anita Białuńska, Anthony P. Salvatore

**Affiliations:** ^1^ Department of Rehabilitation Sciences College of Health Sciences University of Texas at El Paso El Paso TX USA; ^2^ Department of Cognitive Psychology University of Finance and Management in Warsaw Warsaw Poland; ^3^ Department of Communicative Disorders University of Louisiana‐Lafayette Lafayette LA USA

**Keywords:** auditory comprehension, auditory processing, concussion, sensorimotor integration, timing

## Abstract

**Background:**

Although science findings and treatment approaches of a concussion have changed in recent years, there continue to be challenges in understanding the nature of the post‐concussion behavior. There is growing a body of evidence that some deficits can be related to an impaired auditory processing.

**Purpose:**

To assess auditory comprehension changes over time following sport‐related concussion (SRC) in young athletes.

**Methods:**

A prospective, repeated measures mixed‐design was used. A sample of concussed athletes (*n *= 137) and the control group consisted of age‐matched, non‐concussed athletes (*n *= 143) were administered Subtest VIII of the Computerized‐Revised Token Test (C‐RTT). The 88 concussed athletes selected for final analysis (neither previous history of brain injury, neurological, psychiatric problems, nor auditory deficits) were evaluated after injury during three sessions (PC1, PC2, and PC3); controls were tested once. Between‐ and within‐group comparisons using RMANOVA were performed on the C‐RTT Efficiency Score (ES).

**Results:**

ES of the SRC athletes group improved over consecutive testing sessions (*F *=* *14.7, *p *<* *.001), while post‐hoc analysis showed that PC1 results differed from PC2 and PC3 (*ts *≥ 4.0, *ps *< .001), but PC2 and PC3 C‐RTT ES did not change statistically (*t *=* *0.6, *p = *.557). The SRC athletes demonstrated lower ES for all test session when compared to the control group (*ts *> 2.0, *Ps*<.01).

**Conclusion:**

Dysfunctional auditory comprehension performance following a concussion improved over time, but after the second testing session improved performance slowed, especially in terms of its timing. Yet, not only auditory processing but also sensorimotor integration and/or motor execution can be compromised after a concussion.

## 1 INTRODUCTION

Mild traumatic brain injury (mTBI) known as concussion, stands as a prevalent neurotrauma within the general population and has become increasingly common in athletic (Coronado, McGuire, Faul, Sugerman, & Pearson, 2012).

The symptoms of concussion include migraine headaches, cognitive dysfunctions, and neuropsychiatric problems all linked to a complex and variable neuronal pathophysiology (Bolouri & Zetterberg, 2015; Giza & Hovda, 2014). The contributions of oxidative stress and altered neurotransmission, white matter changes, and traumatic axonal injury (Kirov et al., 2013) are thought to be responsible for many of the observed cognitive deficits.

Sports‐related mTBI usually results in symptoms and cognitive deficits that typically resolve within a few days, 5–10 days (Baugh et al., 2016; Giza et al., 2013) or 7–14 days (Eisenberg, Meehan, & Mannix, 2014; Lau, Lovell, Collins, & Pardini, 2009). However, in a subset of athletes, several symptoms and deficits become chronic and can persist even when individuals report they are asymptomatic. Thus, there is high risk for athletes to sustain a second mTBI before symptoms from a first mTBI are resolved if they are permitted to return‐to‐play (RTP) prematurely. Repeated mTBIs among young athletes may be linked to significant neurodegeneration long after retiring from play (Didehbani, Munro Cullum, Mansinghani, Conover, & Hart, 2013; Gavett, Stern, & McKee, 2011).

Post‐concussion neurocognitive tests, used for evaluation of concussed athletes, assess a wide range of cognitive functions including memory, psychomotor speed, attention, impulse control, executive function, and reaction time (RT) (Arrieux, Cole, & Ahrens, 2017). The Immediate Postconcussion Assessment Cognitive Test (ImPACT) (http://www.impacttest.com, 2017) is a commonly used post‐concussion assessment instrument for athletes (Arrieux et al., 2017). However, the sensitivity of this battery for a concussion assessing is a challenge, especially since a concussion can result in a number of different patterns of symptoms and cognitive impairments.

The other indicators of ongoing neuropathology after a concussion are biomarkers such as plasma tau changes (Gill, Merchant‐Borna, Jeromin, Livingston, & Bazarian, 2017) or white matter changes (Narayana, 2017). Recently, Kraus et al. (2016) have shown that the midbrain electrophysiological responses of concussed athletes reflect impaired auditory processing (Kraus, Anderson, White‐Schwoch, Fay, & Popper, 2017). Specifically, the authors demonstrated that mTBI disrupts the processing of the fundamental frequency, a key acoustic cue for identifying and tracking speech, and consequently, understanding speech. They reported that children who sustained a concussion responded with smaller and more sluggish neural responses while tracking fundamental frequency of speech stimuli.

The ability to accurately and efficiently extract meaning from sound is an underpinned by cognitive, sensory, and limbic systems concomitantly. An insult to any of these domains can result in difficulty in understanding of speech (Kraus et al., 2016). Auditory processing is one of the most computationally demanding tasks our brain has to perform. It depends on ongoing, constant changes in sound components. Such fine‐grained analysis relies on multiple neural systems of ascending and descending auditory, sensorimotor, and cognitive networks. Pathophysiological consequences of concussion (e.g., demyelination, axonal injury, or altered neurotransmission) can disrupt the functioning of these systems leading to problems with everyday speech comprehension. Recently, Salvatore, Cannito, Brassil, Bene, and Sirmon‐Taylor (2017) reported that SRC athletes demonstrated deficits in speech understanding. They used Computerized‐Revised Token Test (C‐RTT) as the assessment. It is very likely that these athletes have compromised speech fundamental frequency processing (Kraus et al., 2016), an element of listening in a complex environment.

The Revised Token Test and its computerized version C‐RTT are sensitive tools to detect auditory comprehension impairments as well as improvements in the recovery process in persons with aphasia and children's language impairment (Eberwein et al., 2007; McNeil et al., 2015). The C‐RTT consists of complex tasks which measure speech comprehension of spoken sentences requiring auditory, but also visual and motor processing. Thus, this tool creates more demands on the cognitive processing than mentioned above ImPACT test, and can reflect a disruption in multisensory processing and multisensory integration. For example, Salvatore et al. (2017) showed poor performance of concussed athletes on the C‐RTT as well as on the ImPACT, but results from both tests were related only in terms of delayed motor responses.

In fact, the C‐RTT test, designed for assessing auditory comprehension skills, demands a dynamic combination of sensory information directed at the executions of an intentional motor response (Machado et al., 2010). This process relies on the activity of neural networks, which integrates information from multiple sensory channels that are modulated by the networks communicating between cortical and subcortical areas (Jensen, Kaiser, & Lachaux, 2007). The sensorimotor integration is mediated by attention, emotion, planning, and memory functions and all of these cognitive functions are dysfunctional following a concussion (Arrieux et al., 2017).

The present study was designed to investigate (a) whether sport‐related concussed athletes demonstrate impaired auditory comprehension compared to a group of healthy control (HC) participants; (b) whether dysfunctional auditory comprehension changes over time following a sport‐related concussion; and (c) whether results obtained from CRTT and the ImPACT tests are related in concussed athletes.

We predicted that results of the concussed athletes and the healthy controls athletes would differ; however, the nature and extent of the recovery of auditory comprehension was not clear. We expected that a complex auditory comprehension task that requires multisensory information processing and integration would take longer to recover than visual and verbal memory assessed by the ImPACT test.

## 2 METHODS

### 2.1 Participants

Two groups of participants completed this study, both were convenience samples. The sport‐related concussed group (SRC) consisted of 88 athletes from a sample of 137 athletes ranged from 13 to 38 years of age. Some of the concussed athletes were excluded from the final group for analysis based on their self‐reported brain surgery, psychiatric history, hearing disorder, learning disability, ADD/hyperactivity or dyslexia. The Healthy Control group (HC) was formed by 143 healthy athletes who ranged from 13 to 35 years of age, did not have a self‐reported history of concussion athletes and did not report any history of brain surgery, psychiatric history, hearing disorder, learning disability, ADD/hyperactivity or dyslexia, and were matched for age, sex, and years of education.^1^ Detail information about demographic variables is reported in Table 1. Participants in both groups were recruited from local schools and university community and represented different level of practice, leagues, and a wide range of sports including football, basketball, baseball, ice hockey, soccer, softball, volleyball. Teenagers and young adults participated in high school or university leagues, and the adults were individuals participating in amateur sports.

**Table 1:** Demographic information

	Sport‐related concussed	Healthy control
No. of subjects	88	143
Male sex	64	99
Age, year	18.16 (4.29)	20.41 (3.15)
Total years of educations	10.70 (2.72)	13.01 (2.02)
Previous no. of concussions^a^	0.60 (0.98)	0.17 (0.38)
Symptoms^b^	25.02 (21.81)	3.69 (6.63)

a For HC athletes, if some of them have a concussion history, time since last concussion was always longer than 3 years.

b Total score of concussion symptoms calculated based on Post‐Concussion Symptom Scale included in ImPACT test administered during first/only evaluation (SRC athletes/HC athletes, respectively).

All athletes were referred by trainers or physicians to the Concussion Management Clinic at the University of Texas at El Paso either for baseline testing (HC) or after sustained a sport‐related concussion regardless of perceived severity. All athletes with concussion completed both ImPACT and C‐RTT neurocognitive testing during follow‐up three times (PC1, PC2, and PC3). Clinical management and care of participants followed international RTP standards (McCrory et al., 2013). Athletes were followed clinically not according to a controlled research protocol. The general course was to conduct the first evaluation within 72 hours, then 2 and 3 weeks after injury. The median time to first evaluation was 4 days (mea*n *= 5.9 days, *SD* = 6.2), second evaluation 11 days (mea*n *= 13.2 days, *SD* = 12.4), and third 20 days (mea*n *= 26.8 days, *SD* = 21.6). HC athletes were evaluated once using the same protocol as used for SRC patients.

Before testing, the Institutional Review Board approval and written informed consent from subjects were obtained.

### 2.2 Evaluation

The current study used two neurocognitive assessment software instruments: ImPACT test version 2 and C‐RTT Subtest VIII, which were administered in that order. ImPACT is a neurocognitive battery, composed of three sections: a demographic/health questionnaire, the 22–item Post‐Concussion Symptom Scale (PCSS; separated into four clusters—migraine, cognitive, neuropsychiatric, and sleep (Lovell et al., 2006)) based on 7‐point Likert‐type scale, and six neurocognitive test modules, evaluating different aspects of attention, memory, processing speed, and reaction time. For the individual tests and the construction of composite scores, see Schatz, Pardini, Lovell, Collins, & Podell (2006). The ImPACT test takes approximately 25 min to complete and uses a computer monitor and mouse to present and respond to the test stimuli.

**Figure 1:**
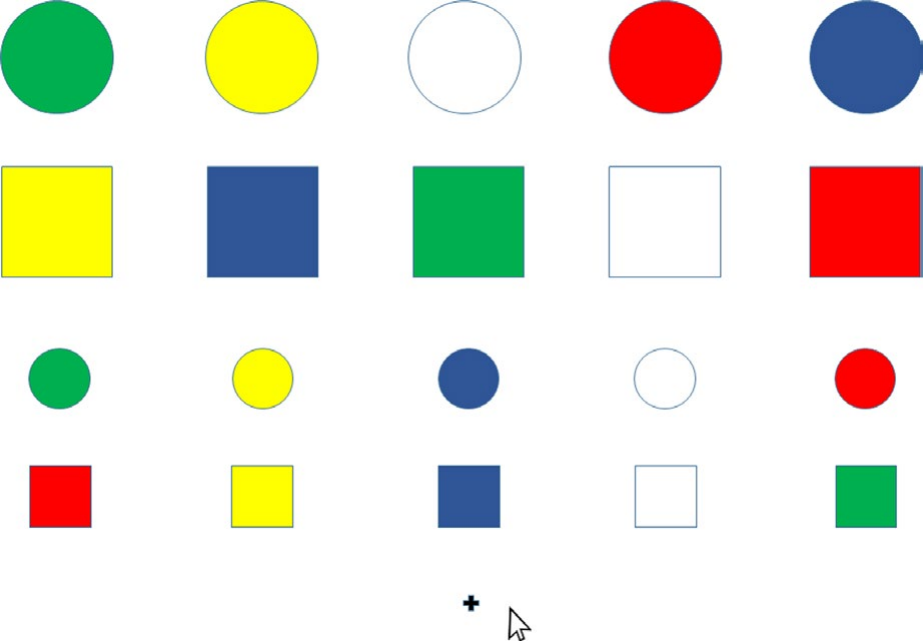
An example of the item from the Subtest VIII of the C‐RTT. Such board is presented on a screen and is accompanied by the aurally presented instruction: “Put the small, yellow circle to the left of the big, red square”

The second test was Subtest VIII of the C‐RTT test, a computerized version of the Revised Token Test (RTT) (McNeil & Prescott, 1978). This test is a standardized clinical research tool to assess the severity and the nature of auditory language processing deficiencies linked to brain damage such as aphasia, language and learning disabilities, and recently mTBI/concussion (Salvatore et al., 2017). This test consists of 10 subtests varying in stimuli complexity and task difficulty, but in all of them, listeners are asked to manipulate objects varying in size, shape, and color in accordance with sentence meaning. Comprehension of the sentences is demonstrated by having the listener touch the correct tokens and put them in the particular position relative to another with a cursor (see Figure 1). Subtest VIII of the C‐RTT is the most difficult subtest. The test assesses the accuracy and speed of an individual's responses to structurally and informationally complex spoken sentences. The digitally recorded sentences are presented by C‐RTT software and participants respond to the sentences by manipulating tokens presented on a computer screen via a mouse. The C‐RTT uses a multidimensional, automatic scoring system (Eberwein et al., 2007; Salvatore et al., 2017). Subtest VIII of the C‐RTT presents 10 sentences and requires about 10 min to be completed.

### 2.3 Statistical analysis

All statistics were performed using IBM SPSS^®^ Statistics Version 23. A mixed‐design was used with an inception cohort study (prognosis). Means and standard deviations were calculated for all output scores. The variables of interest were as follows: three measures derived from the Subtest VIII of the CRTT: accuracy (A), reaction time (RT), and the efficiency score (ES) automatically calculated by the software based upon a ratio between accuracy and response time. The maximum accuracy score of 15 reflected an accurate and prompt response initiated during the average sentence duration of 4.26 s. The additional cognitive measures produced by the ImPACT test were as follows: Visual Memory Composite, Verbal Memory Composite, Visual Motor Speed Composite, and Impulse Control, and Reaction Time. We conducted between‐ and within‐group comparisons. Each PC outcome was compared to HC outcome by independent sample *t* or *Friedman* test. Results of the SRC athletes collected over three PC evaluations were entered in a series of mixed‐design ANOVAs, for *PC, age*,* school*, or *sex* within‐subject factors and *Group* between‐subject factor. Differences between each outcome results were tested with Bonferroni‐corrected *t*‐tests (*p *<* *.05).

## 3 RESULTS

The average values of the variables of interest as a function of time of evaluation (PC1, PC2, and PC3) obtained in SRC athletes, as well as results obtained in HC group, are presented in Table 2.

**Table 2:** Sport‐related concussed (SRC) and Healthy control (HC) Athletes groups means (SD) for Each Outcome Variable recorded during Three Consecutive Post‐Concussion Evaluations (PC1, PC2, and PC3)

Variable	Sport‐related concussed (*n *= 88)			Healthy control (*n *= 143)
	PC1	PC2	PC3	
CRTT				
Accuracy (A)	14.09 (0.79)	14.19 (0.74)	14.45 (0.58)	14.47 (0.60)
Reaction Time (RT)	2543.59 (969.88)	2298.36 (769.03)	2150.67 (542.04)	1997.17 (534.90)
Efficiency (E)	12.15 (1.05)	12.61 (0.88)	12.66 (0.58)	12.85 (0.63)
ImPACT				
Verbal Memory	81.66 (12.61)	85.19 (9.43)	88.48 (8.31)	89.11 (8.40)
Visual Memory	69.31 (14.27)	70.69 (12.77)	76.30 (9.84)	78.56 (10.31)
Visual Motor Speed	33.67 (7.09)	35.38 (6.20)	37.06 (6.49)	39.87 (6.58)
Reaction Time	0.67 (0.17)	0.60 (0.10)	0.58 (0.08)	0.58 (0.07)
Impulse Control	5.51 (4.12)	5.73 (4.28)	5.33 (4.26)	4.25 (3.45)

First, to test whether the SRC athletes demonstrate impaired auditory comprehension as compared to a matched HC group across time period, results obtained in the Subtest VIII of CRTT from the each evaluation at PC1, PC2, and PC3 were compared separately to the HC athletes’ performances using *t*‐tests or nonparametric independent two‐samples test, respectively, considering a level of measurement.

SRC athletes demonstrated lower ES and longer RT at PC1, PC2, and PC3 as compared to HC group (ES: *t* = 5.6, *p *<* *.001; *t* = 2.2, *p* = .030; *t* = 2.2, *p* = .029; RT: *t *=* *4.8, *p *<* *.001; *t *=* *3.2, *p *=* *.002; *t *=* *2.1, *p *=* *.036). However, CRTT performances’ accuracy scores of SRC athletes and HC athletes differed only comparing results of PC1 and PC2 evaluations (*Z *=* *2.2, *p *<* *.001 and *Z *=* *1.5, *p *=* *.028), but not PC3 (*Z *=* *0.6, *p *=* *.838).

Next, to verify whether auditory comprehension changes over time following a concussion, RT and ES data from three PC evaluations of SRC athletes obtained in Subtest VIII of CRTT were entered to the repeated‐measure analysis of variance. The Testing Session (PC1, PC2, and PC3) was the within‐subject factor; concussed athletes were taken as the random variable. To determine whether the effect of three PCs evaluation of the auditory comprehension on A was significant, the Friedman test was used.

ES of SRC improved over consecutive PC evaluations (*F *=* *14.7, *p *<* *.001), post‐hoc showed that PC1 results significantly differed from PC2 and PC3 (*t *=* *4.0, *p *<* *.001 and *t *=* *4.9, *p *<* *.001), but PC2 and PC3 did not differ within the group (*t *=* *0.6, *p = *.557). Furthermore, as can be seen in Table 2, RT decreased over consecutive PC test sessions, giving a significant main effect of testing session (*F *=* *10.6, *p *<* *.001), and significant differences between PC1 vs. PC2, PC2 vs. PC3, and PC1 vs. PC3 (*t *=* *2.7, *p *<* *.001; *t *=* *2.1, *p = *.042 and *t *=* *4.1, *p *<* *.001). Moreover, the A changes over time after SRC tested by Friedman Test also reached significance (χ^*2*^=24.1, *p *<* *.001). As can be seen in Table 2, SRC athletes’ accuracy performances on CRTT Subtest VIII systematically improved over test sessions (post‐hoc PC1 vs. PC2: *Z *=* *2.9, *p *=* *.003; PC2 vs. PC3: *Z *=* *2.2, *p *=* *.028, and PC1 vs. PC3: *Z *=* *4.9, *p *<* *.000).

We also verified whether age, a level of education, and sex were related to these changes. The CRTT data from SRC athletes were submitted into the mixed‐design ANOVAs, separately for RT and ES, considering athletes as the random variable.^2^ Groups isolated based on age, a level of education, and sex were the between‐subjects factors and time of evaluation (PC1, PC2, and PC3) was the within‐subjects factor. Neither age, nor years of education, nor sex did not affect changes in ES and RT performance over time. No interaction of two factors: time of evaluation and age or level of education or sex was not significant (*F *=* *0.1, *p *=* *.988; *F *=* *0.5, *p *=* *.686 and *F *=* *1.9, *p *=* *.164 for ES; and *F *=* *1.0, *p *=* *.403; *F *=* *0.5, *p *=* *.702 and *F *=* *0.6, *p *=* *.546 for RT).

Finally, to test whether results obtained from C‐RTT and the ImPACT test could lead to different evaluation of concussed athletes, we analyzed group differences in ImPACT data collected during the same three evaluation times as C‐RTT data. Specifically, the five scores on the ImPACT test of the three test sessions were separately compared to the ImPACT results of the HC groups of athletes. Results show that both groups at test session one differ for all ImPACT scores. The SRC group performed poorer than the HC group across all scores. At the first post‐concussion test session, these athletes generated lower average Visual Memory Composite (*t *=* *5.1, *p *<* *.001), Verbal Memory Composite scores (*t *=* *4.7, *p *<* *.001), Visual Motor Speed Composite score (*t *=* *6.5, *p *<* *.001), higher average Impulse Control score (*t *=* *2.3, *p *=* *.021), and average Reaction Time (*t *=* *4.2, *p *<* *.001) than the HC group. At the second post‐concussion test session, the SRC athletes were still showed poorer Visual Memory Composite scores (*t *=* *4.7, *p *<* *.001), Verbal Memory Composite scores (*t *=* *3.2, *p *=* *.002), Visual Motor Speed scores (*t *=* *5.0, *p *<* *.001), and average Impulse Control scores (*t *=* *2.7, *p *=* *.009) than the HC group. However, there was no significant effect of group on average Reaction Time at the second test session (*t *=* *1.3, *p *=* *.195). At the third testing session, the SRC athletes and the HC group continued to show statistically significant differences for Visual Motor Speed score (*t *=* *3.1, *p *=* *.002). The other four scores were not significantly different between the two groups (Visual Memory Composite score *t *=* *1.6, *p *=* *.113, Verbal Memory Composite score *t *=* *0.5, *p *=* *.590; average Reaction Time *t *=* *0.7, *p *=* *.460; Impulse Control score *t *=* *1.9, *p *=* *.060).

## 4 DISCUSSION

The purpose of this study was to evaluate changes in auditory comprehension performance after experiencing a sport‐related concussion. The test used can be treated as a measure of auditory processing of complex speech utterances, but also as a tool that assesses multisensory integration, motor preparation, and execution.

Performance on the C‐RTT demonstrated that auditory comprehension of spoken sentences is dysfunctional in athletes with SRC confirming previous findings by Salvatore et al. (2017). The problems are manifested in delayed and less accurate motor responses to spoken sentences when compared to an HC group of athletes. This effect is not due to differences in age, years of education, and gender. Efficiency Score performance on the C‐RTT improves over time following a concussion, but mostly from first to second test session. About 11 days after injury, the recovery slowed. Thus, 3 weeks after injury, the SRC and HC accuracy on the C‐RTT did not differ, but reaction time was still slower for the SRC group. However, the HC and SRC groups’ performance on the ImPACT scores showed no statistically significant differences at test session three.

What mechanisms are responsible for auditory comprehension dysfunctions and why recovery of them showed different course than other cognitive impairments? What does finding mean in clinical context? Each concern is discussed in turn.

Different hypotheses can account for the effect of auditory comprehension perturbation after mTBI. The auditory processing of spoken utterances relies on the successful integration of information across the auditory system of distributed, integrated circuit of cognitive, sensorimotor, and rewards neural pathways (Kraus & White‐Schwoch, 2015). These networks process acoustic cues conveyed by auditory signals across timescales into meaningful percepts. The insensitivity to temporal cues at one or more rates (corresponding to phonemes, syllables, sentences decoding) as a result of the insult to any component of the auditory brain networks may compromise the ability to understand speech. It should come as no surprise that a biomechanical force to the brain will result in either functional (cellular ionic shifts, metabolic changes, or impaired neurotransmission) or microstructural (axonal) injury to neural tissue resulting in a dysfunction in auditory processing (Giza & Hovda, 2014). Kraus et al. (2016) recently reported that a concussion disrupts the processing of the fundamental frequency of speech (F_0_). They recorded speech‐evoked FFRs, the product of summary neural activity in the auditory midbrain that reflects the coding of speech features. Thus, dysfunctional auditory comprehension in athletes with a concussion may be a consequence of impaired F_0_ processing mechanisms. Although this hypothesis is plausible, it is unlikely that disruption of F_0_ processing can explain completely C‐RTT results obtained in this study. It is well documented that the strength of coding of the F_0_ in speech underlies successful speech understanding in noise (Anderson, White‐Schwoch, Parbery‐Clark, & Kraus, 2013). In contrast, participants in the present study were tested in a sound‐treated room, using a comfortable listening level, determined by each participant. Kraus et al. (2016) showed that F_0_ improves as concussion symptoms resolve. Yet, our findings showed that although C‐RTT performances of concussed athletes improved over time, they still performed poorer than the HC group during all three test session.

Another possible explanation for the documented dysfunction is that other brain mechanisms may contribute to processing auditory stimuli following a concussion. Listening engages cognitive networks and the precision of sound processing is linked to cognitive skills as attention and working memory (Kraus et al., 2016). These processes activate neural circuits within temporal and frontal cortices (Stanley et al., 2015), which are the areas of neocortex most susceptible to injury in a concussion (Narayana, 2017). Also, multimodal MRI studies identified that corpus callosum (CC), particularly splenium, is the most commonly affected white matter tract in mTBI (Aoki, Inokuchi, Gunshin, Yahagi, & Suwa, 2012) and that CC white matter changes can be associated with graded deficits in working memory (Prins, Hales, Reger, Giza, & Hovda, 2011). Thus, reduced C‐RTT responses of athletes with concussion can be due to the attentional or working memory systems dysfunctions, which no longer sufficiently facilitate speech processing. An additional argument may strengthen this hypothesis is that responses to the second half of command are less accurate than responses to the first phrase in the test sentences (Salvatore et al., 2017). This finding may indicate that working memory is overloaded after the first part of the sentence is being held for processing. The second part is not being integrated into the processing of the first phrase and thus is degraded leading to more errors for the second phrase (Salvatore et al., 2017). Interestingly, our findings show different patterns of recovery for visual and verbal memory, and attention, as measured by the ImPACT test, compared to the recovery of auditory comprehension as assessed by C‐RTT. This contrast in performance questions the position the auditory language processing dysfunction after mTBI is the consequence of attentional or/and working memory dysfunctions.

An important novel finding of this study was that even accuracy of motor responses to spoken sentences improved over time following a concussion, such processing is still impaired in terms of timing compared to HC group's response time. Improvement of the responses timing on the C‐RTT test in the SRC group differs in comparison to the ImPACT scores: visual and verbal memory, attention, and impulse control. It is likely, that neural timing mechanisms, which rely on neural circuits partially sharing auditory and cognitive processing networks in the temporal and prefrontal cortex, are impaired after a concussion. The impaired reaction time and processing speed which are the most robust findings in studies predicting concussion status support such hypothesis (Arrieux et al., 2017). This explanation is compatible with the assumption that the auditory system following a concussion is impaired. In fact, extract meaning from sounds relies on high temporal resolution tracking the changes of key acoustic features across timescales. Thus, dysfunctions of timing within auditory modality and speech domain can be more evident than in vision. Auditory modality dominates in time information processing over visual modality and contrary visual modality dominates over audition in space information processing (Repp & Penel, 2002). Underpinnings of timing processing involve at least two functionally distinct systems, implicating separate, but integrated cortical–subcortical networks including a motor system (cerebellum and primary and secondary motor cortices), and a cortical–subcortical loop (basal ganglia, parietal cortex, and prefrontal areas) (Repp & Su, 2013). The insult to any of these components, as a result of post‐concussion pathophysiology including deficits in excitatory mechanisms (N‐methyl‐D‐aspartate receptors) relevant for cortical connections and information transmission (Giza & Hovda, 2014) and white matter disruption in CC and subcortical white matter (Narayana, 2017), is plenty possible and may compromise timing processing.

However, Martini, Eckner, Meehan, and Broglio (2017) evidenced that adolescents who have sustained a concussion do not show impaired performance on a discrete temporal auditory task. We note, however, that this investigation had some limitations in arguing that the children showed actual timing deficits after a concussion. The authors analyzed only timing variability. We recognize that other timing features such as accuracy can also be disrupted. They used metronomic task: a pacing tone was played at either a 1 Hz or 0.5 Hz rate for a total 60 s and participants responded as soon as each tone was presented. Isochronous sequence automatically trigger expectation of the moment of tones occurrence (Repp & Su, 2013) and it is hard to respond to stimulus not to synchronize with. On the other hand, intervals between consecutive sounds either 1 Hz or 0.5 Hz rate exceed typical tempo of synchronization tasks. In fact, synchronization to sounds presented every 2 s is impossible and participants rather respond to them. However, if participants use subvocal counting, it may enable and facilitate synchronization with tones presented even around 0.5 Hz rate. People with at least basic music experience usually use such strategy of mental subdivisions performing synchronization tasks.(Repp, 2010) The authors do not report the eventual musical experience of tested participants and it is uncertain what task was exactly assessed in that study. The different timing mechanisms are involved in time processing depending on tasks. Białuńska, Dalla Bella, and Jaskowski (2011) showed that manipulation of stimulus intensity affected RT, but did not affect precision in the synchronization task. Stimulus predictability may impinge on the functioning of additional timing mechanisms facilitating performances. So timing in the metronomic task may not be enough sensitive measure of timing system dysfunctions.

Another possible reason for the reduced C‐RTT's performances can be a dysfunction of sensorimotor integration. The coordination of multisensory information for the control of movement is the fundamental aspect of the sensorimotor process and requires constant change of different elements of the nervous system occur in the subcortical and cortical sites, neurotransmitters, synapse gaps, neuron ramification, and variety of other micro‐ and macrostructures in the brain sometimes very distant (Velasques et al., 2011). Slobounov et al. (2011) reported disrupted brain connectivity in concussed athletes, which may affect the integrity of the CC and integration of information processing in distinct networks (van den Heuvel & Sporns, 2013).

Based on data collected in presented study, it is hard to distinguish which mechanisms are primarily responsible for observed dysfunctional speech processing in terms of delayed motor responses. It required further investigation using different methods of assessment, biologic markers together with cognitive tests. The described dissociations of cognitive functioning restoration, as assessed by various neurocognitive tools, require different levels of multisensory processing, pointed rather at more general mechanisms impairment, responsible for timing or sensorimotor integration.

Our findings are unique for another reason. If the recovery after concussion across different function within neural system seems to be steady and similar during the initial phase after injury, but then begins to dissociate and some simple function continue the course of restoration, but the others slow down, it might be a clue for a different model of concussion management than is currently recommended (Giza et al., 2013). It is likely that after acute phase some functional, metabolic perturbations to neural tissue calm down by resting, and some post‐concussed impairments mitigate and tend to be completely resolved during the acute phase of concussion. However, resting may be not the best recommendation for enabling other cognitive dysfunctions restoration. It might be that some more severe, permanent consequences of microstructural axonal injury or altered neurotransmission, like dysfunctions in coding acoustic cues during speech processing or impaired timing needed for neuroplastic changes. Sensory systems have astonishing ability to reshape response properties following learning, and in the auditory system, plasticity has been observed from cochlea to cortex. However, it requires specific training and rich stimulation (Kraus & White‐Schwoch, 2015). For example, both timing and sensorimotor integration improve based on music practice experience. Dalla Bella et al. (2017) proved that impaired gait and timing in patients with Parkinson disease improve after rhythmic auditory stimulation training.

The study is not without limitations. It was observational, and this methodology did not allow experimental factors such as standardized testing intervals. The athletes were returned to play when they met clinical criteria, not a specific time after injury, thus leading to variable testing intervals. In both groups, there was a predominance of male, and although groups were age‐matched, distribution of participants was different.

Summarizing, the athletes with SRC exhibit auditory comprehension deficits which improved over time following a concussion, however, the gain of auditory comprehension showed a longer recovery period than the cognitive functions measured by the ImPACT test and persistence of symptoms. As we continue to understand the pathophysiology of sports concussion and its implications better (Iverson, Brooks, Collins, & Lovell, 2006), there is increasing scrutiny to manage the injury better. This study shed light that acutely and chronically recommendations for concussion management may be better compiled considering neurocognitive testing of multisensory processing, not only via the visual modality.

## Footnotes

1 Independent sample tests (*t* and χ^*2*^ ) were used to compare demographic variables between both groups.

2 For all ANOVAs’ results reported in this paper, the Greenhouse–Geisser correction for inhomogeneity of variance was applied whenever appropriate.
